# Effect of moderate altitude and nocturnal oxygen therapy on cerebrovascular function in patients with COPD: A randomized, crossover trial at 2048 m

**DOI:** 10.1113/EP093003

**Published:** 2025-07-22

**Authors:** Dominic Gilliand, Tsogyal D. Latshang, Sayaka S. Aeschbacher, Fabienne Huber, Deborah Flueck, Mona Lichtblau, Stefanie Ulrich, Elisabeth D. Hasler, Philipp M. Scheiwiller, Julian Müller, Silvia Ulrich, Konrad E. Bloch, Michael Furian

**Affiliations:** ^1^ Department of Respiratory Medicine University Hospital of Zurich Zurich Switzerland; ^2^ Research Department Swiss University of Traditional Chinese Medicine Bad Zurzach Switzerland

**Keywords:** cerebrovascular reactivity, chronic obstructive pulmonary disease, hypoxia, transcranial Doppler ultrasound

## Abstract

We investigated whether nocturnal oxygen therapy improves next‐day cerebrovascular function in lowlanders with chronic obstructive pulmonary disease (COPD) staying at moderate altitude. This randomized, placebo‐controlled single‐blind crossover trial was performed in moderate‐to‐severe COPD patients [forced expiratory volume in 1 s (FEV_1_)/forced vital capacity (FVC) <0.7; FEV_1_ 30%–80% of predicted], living at <800 m a.s.l. and arterial oxygen saturation ($S_{pO_{2}}$) measured with pulse oximetry ≥92%. Patients underwent assessments at 490 m and during two separate sojourns of 2 days at 2048 m, receiving either 3 L min^−1^ nocturnal oxygen therapy or placebo in a randomized crossover design. At both altitudes, $S_{pO_{2}}$, cerebral tissue oxygenation (CTO, measured by near‐infrared spectroscopy), mean arterial blood pressure (MAP, measured by finger plethysmography) and middle cerebral artery systolic peak blood flow velocity (sMCAv, measured by transcranial Doppler ultrasound) were assessed while patients were quietly breathing with fraction of inspired O_2_ ($F_{IO_{2}}$) 0.21, with $F_{IO_{2}}$ 1.0, voluntarily hyperventilating, voluntarily hyperventilating with $F_{IO_{2}}$ 1.0, and during a head‐up tilt. Overall, 18 patients (8 women) aged (mean ± SD) 65 ± 5 years, with FEV_1_ 54.7% ± 13.9% predicted were analysed. At 2048 m under $F_{{\mathrm{IO}}_{2}}$ 0.21, patients became hypoxaemic ($S_{pO_{2}}$ 90.3% ± 1.6%), while MAP, CTO and sMCAv remained unchanged compared with 490 m. All ventilatory manoeuvres at 2048 m induced greater increases in $S_{pO_{2}}$ compared with 490 m, while changes in MAP, CTO and sMCAv were similar. Head‐up tilt induced a similar decrease in blood pressure, whereas sMCAv changed less in response to systemic hypotension (ΔsMCAv/ΔMAP 0.9 ± 1.3 vs. 2.3 ± 1.7 cm s^−1^ mmHg^−1^) at 2048 m. No effect of nocturnal oxygen therapy was observed during any manoeuvres. This randomized clinical trial in moderate‐to‐severe COPD patients ascending to 2048 m showed that moderate hypoxaemia does not translate to daytime cerebral hypoxia or cerebrovascular autoregulatory impairments while at rest or during ventilatory or orthostatic challenges.

## Abstract (highlights)

- **What is the central question of this study?**
  Patients with chronic obstructive pulmonary disease (COPD) develop altitude‐related illnesses and experience disturbed sleep and cerebrovascular autoregulation disturbances during physical exertion at moderate altitude. Is cerebrovascular homeostasis and responsiveness in COPD impaired in resting conditions at moderate altitude? Does nocturnal oxygen therapy improve cerebrovascular function?
- **What is the main finding and its importance?**
  On the first days at 2048 m, resting cerebrovascular function remained intact in COPD patients. Although nocturnal oxygen therapy improved nocturnal indices and prevented altitude‐related illnesses, no beneficial effect on next‐day cerebrovascular homeostasis and reactivity at 2048 m was detected.

## 1 INTRODUCTION

Chronic obstructive pulmonary disease (COPD) is a systemic disease characterized by reduced lung function and airflow obstruction and is one of the world's leading causes of death. Given the high prevalence of COPD of 8%–15% of the adult world population and recent improvements in symptom‐mediated treatments, it is assumed that many COPD patients are among individuals living at low altitude but ascending to high altitude for work or recreational activities (Global Initiative for Chronic Obstructive Lung Disease, Inc., 2020). Acute exposure to hypobaric hypoxia at higher elevations can pose a challenge for an impaired respiratory system with less ventilatory reserve (Bloch et al., 2023). Previous studies have shown that some patients with stable moderate‐to‐severe COPD develop altitude‐related illnesses (Furian et al., 2022; Furian, Lichtblau et al., 2018; Tan et al., 2020) and experience exercise intolerance, disturbed sleep and disturbed breathing patterns at altitude (Furian, Flueck et al., 2018; Gutweniger et al., 2021; Latshang et al., 2019; Scheiwiller et al., 2022). Moreover, at moderate altitude (2048 m), cerebrovascular autoregulation was unable to protect the brain from cerebral oxygen desaturations while sleeping and during physical exertion (Gutweniger et al., 2021; Tan et al., 2020). Whether cerebrovascular homeostasis and responsiveness in COPD are impaired in quiet daytime conditions at moderate altitude has not been studied.

In a randomized clinical trial investigating the preventive effect of nocturnal oxygen therapy (NOT) in COPD at moderate altitude, mixed effects were observed. Tan and colleagues (2020) studied the effect of NOT on altitude‐associated periodic breathing during sleep at 2048 m. They found an improvement in nocturnal arterial and cerebral oxygenation and stabilized breathing patterns at night compared with placebo. Furthermore, the participants had significantly improved subjective sleep quality and experienced fewer high‐altitude‐related illnesses. Within the same trial, Meszaros et al. (2021) have shown that NOT mitigated the altitude‐induced rise in systolic blood pressure (BP) and elevated baroreflex sensitivity compared with placebo at altitude. Whether NOT improves cerebrovascular function the next morning at physical rest remains to be elucidated.

Therefore, the aim of this study was to investigate altitude‐related changes in the cerebrovascular homeostasis and responsiveness in resting conditions and to elucidate the effects of NOT in patients with COPD staying overnight at moderate altitude.

## 2 MATERIALS AND METHODS

### 2.1 Ethical approval

The trial was approved by the cantonal ethics committee of Zurich (EK‐2013‐0088) and conformed to the latest revision of the *Declaration of Helsinki*. The main trial was registered at clinicaltrials.gov with the identifier NCT02150590. All participants gave informed written consent.

### 2.2 Study design and intervention

This study is part of a randomized, placebo‐controlled single‐blind crossover trial performed at the University Hospital of Zurich (490 m) and at St Moritz (2048 m) to evaluate the effect of NOT for COPD patients on nocturnal hypoxaemia, altitude‐associated periodic breathing and altitude‐related illnesses. The outcomes above have been published previously (Tan et al., 2020). Other findings of this study have been published elsewhere (Bisang et al., 2021; Gutweniger et al., 2021; Lichtblau et al., 2020; Meszaros et al., 2021; Tan et al., 2020), whereas the present findings related to cerebrovascular homeostasis and responsiveness have not been published.

Participants performed baseline measurements at 490 m and during two sojourns at 2048 m for a period of 2 days and 2 nights per stay with a washout period of 2 weeks in between. At 2048 m, participants were allocated randomly to receive either NOT (100% oxygen, $F_{IO_{2}}$ 1.0) or placebo (ambient air, $F_{IO_{2}}$ 0.21) at 3 L min^−1^ flow rate via nasal cannula during the night and were blinded to this intervention. Owing to study safety rules and ethical considerations, the nocturnal arterial oxygenation of patient was monitored by the investigators, hence they were not blinded to the intervention. Transfers between 490 and 2048 m were made by train and car within 3 h. Usual medication was continued during the trial.

### 2.3 Participants

Eligible patients of either sex, diagnosed with moderate to severe COPD according to the GOLD criteria [with forced expiratory volume in 1 s (FEV_1_)/forced vital capacity (FVC) < 0.7 and FEV1 between 30% and 80% of predicted] (Global Initiative for Chronic Obstructive Lung Disease, Inc., 2020), aged 18–75 years and living at <800 m a.s.l., were invited to participate in this study. Criteria for exclusion were past exacerbation of COPD within 4 months before the trial, oxygen saturation of <92% at 490 m, any uncontrolled cardiovascular disease, routine day or night supplemental oxygen therapy at altitude of residence, use of continuous or bilevel positive airway pressure devices, previous altitude intolerance or altitude exposure >1500 m for >2 days within the last 4 weeks before the trial. During the trial, patients were withdrawn from the study for safety reasons if they experienced one of the following predefined altitude‐related adverse health effects: systolic or diastolic BP >200 mmHg or >110 mmHg, respectively; $S_{pO_{2}}$ <75% at rest for >30 min; acute chest pain at rest; acute mountain sickness; COPD exacerbation; or any other condition requiring medical treatment or relocation to low altitude.

### 2.4 Manoeuvres to assess cerebrovascular function

At 490 m and on the second day at 2048 m, patients underwent cerebrovascular testing in calm supine conditions while performing instructed breathing manoeuvres, followed by a supine‐to‐60° head‐up tilt. The breathing manoeuvres aimed to produce different states of arterial partial pressure of CO_2_ and O_2_. Cerebral blood flow (CBF) was assessed during the following conditions with the breathing manoeuvres intended to induce a respective intensification of cerebral vasoconstriction: first, in resting condition, poikilocapnic hypoxia (in respect to variable CO_2_‐levels) with quiet room air breathing ($F_{{\mathrm{IO}}_{2}}$ 0.21); second, poikilocapnic hyperoxia with quiet oxygen breathing ($F_{IO_{2}}$ 1.0); third, hypocapnic normoxia withambient air hyperventilation; and forth, hypocapnic hyperoxia with oxygen hyperventilation. In a specified sequence cycle, participants were asked to carry out 10 min of quiet breathing, followed by an episode of hyperventilation until a plateau of the end‐tidal partial pressure of CO_2_ ($P_{\mathrm{ET},CO_{2}}$) was reached for ≥15 s. This sequence was carried out twice in a row while either ambient air ($F_{IO_{2}}$ 0.21) or oxygen ($F_{IO_{2}}$ 1.0) was administered to the participants via a face mask with a reservoir bag. The order of ambient air or oxygen administration for the breathing manoeuvres was randomized. After the breathing manoeuvres, a washout period of ≥10 min was allowed for normalization of all parameters. Finally, a head‐up tilt was applied by inclining the bed to 60° (ergoline GmbH, Bitz, Germany) for 3 min to provoke a rapid reduction of mean arterial blood pressure (MAP) and consecutive vasodilation. All measurements were performed in the morning.

### 2.5 Measurements

During all manoeuvres, continuous variable measurements were performed. Peak systolic blood flow velocity of the middle cerebral arteries (sMCAv) was measured by transcranial Doppler ultrasound (TOCM, Multigon Industries, Yonkers, NY, USA) with 2 MHz probes placed bilaterally at the temporal windows. The position and settings were identical at each session within a patient. Beat‐by‐beat blood pressure and heart rate were measured non‐invasively with the finger‐cuff technique (Finapres Midi, FMS, The Netherlands) with the finger positioned at heart level. Systolic and diastolic blood pressure values of the finger were calibrated by the mean of three brachialis sphygmomanometer measurements. Breath‐by‐breath $P_{\mathrm{ET},CO_{2}}$ was assessed by capnography (Capnocheck Sleep; Smiths Medical PM Inc., Waukesha, WI, USA). Additional measurements included pulse oximetry ($S_{pO_{2}}$) and cerebral near‐infrared spectroscopy (NIRO 200NX device, Hamamatsu, Japan) to assess cerebral tissue oxygenation (CTO), oxygenated (O_2_Hb) and deoxygenated (HHb) haemoglobin concentrations as surrogates for changes in local cerebral oxygenation, and total haemoglobin concentration (totHb) as an index of local cerebral blood volume.

Averages of all parameters were calculated using data that were measured during predefined time periods. Measurements of the quiet breathing manoeuvres were conducted during the last 3 min of the period at rest, with a 30 s gap before the start of hyperventilation, and the last 15 s of the $P_{\mathrm{ET},CO_{2}}$ plateau during the hyperventilation manoeuvres was analysed.

### 2.6 Outcome calculation

Cerebrovascular responsiveness to blood gases was assessed as a change of sMCAv per unit change of $P_{\mathrm{ET},CO_{2}}$ (ΔsMCAv/Δ$P_{\mathrm{ET},CO_{2}}$; in centimetres per second per millimetre of mercury) and $S_{pO_{2}}$ (ΔsMCAv/Δ$S_{pO_{2}}$; in centimetres per second per percentage) (Hartmann et al., 2015).

During the time period of the head‐up tilt, dynamic cerebral autoregulation was quantified by the total unit reduction of sMCAv per unit reduction of MAP (ΔsMCAv/ΔMAP; in centimetres per second per millimetre of mercury). The percentage reduction of MCAv per unit reduction of MAP (%ΔsMCAv/ΔMAP; as a percentage per millimetre of mercury) and the percentage reduction of sMCAv per percentage reduction of MAP (%ΔsMCAv/%ΔMAP; unitless), with higher values indicating more impaired autoregulation (Numan et al., 2014). Furthermore, the cerebrovascular conductance index (CVCi = sMCAv/MAP; in centimetres per second per millimetre of mercury) and its reciprocal, the cerebrovascular resistance index (CVRi = MAP/sMCAv), were calculated for each manoeuvre, assuming a stable vessel diameter.

### 2.7 Outcomes

The main outcomes were the variables reflecting changes in sMCAv in response to $P_{\mathrm{ET},CO_{2}}$, $S_{pO_{2}}$ and MAP alterations as described in section 2.6. Secondary outcomes included the CVCi and CVRi, and the changes of parameters obtained from near‐infrared spectroscopy for each manoeuvre, respectively.

### 2.8 Statistical analysis

All data were analysed using linear mixed regression models. Model fitting was tested with fitted residuals and by visually inspecting the model residual distribution using kernel density plots. In the event of normal distribution, data were summarized by the mean ± SD; for non‐normal distribution of the residuals, data were summarized by the median (interquartile range). Altitude‐ or intervention‐induced differences were calculated and presented as mean differences [95% confidence intervals (CI)] using mixed linear regression models with patients as random effects and with altitude (490 or 2048 m), nocturnal intervention (NOT or placebo) and cerebrovascular challenges and their interaction as fixed effects. A value of *p *< 0.05 reflected a statistically significant difference. Data analysis was performed in the per‐protocol population, defined as patients undergoing all scheduled assessments and with both measurements for MAP and sMCAv successfully performed within the required time frame for at least one of the four manoeuvres. Statistical analysis was performed in STATA v.15.

Sample size estimation was performed for the main outcome of the randomized, placebo‐controlled single‐blind crossover trial evaluating the effect of NOT on nocturnal hypoxaemia and the apnoea–hypopnoea index (Tan et al., 2020). For this subproject, no sample size estimation has been performed, mainly owing to the unknown threshold for a clinically relevant change in sMCAv.

## 3 RESULTS

### 3.1 Study flow and patient characteristics

**FIGURE 1:**
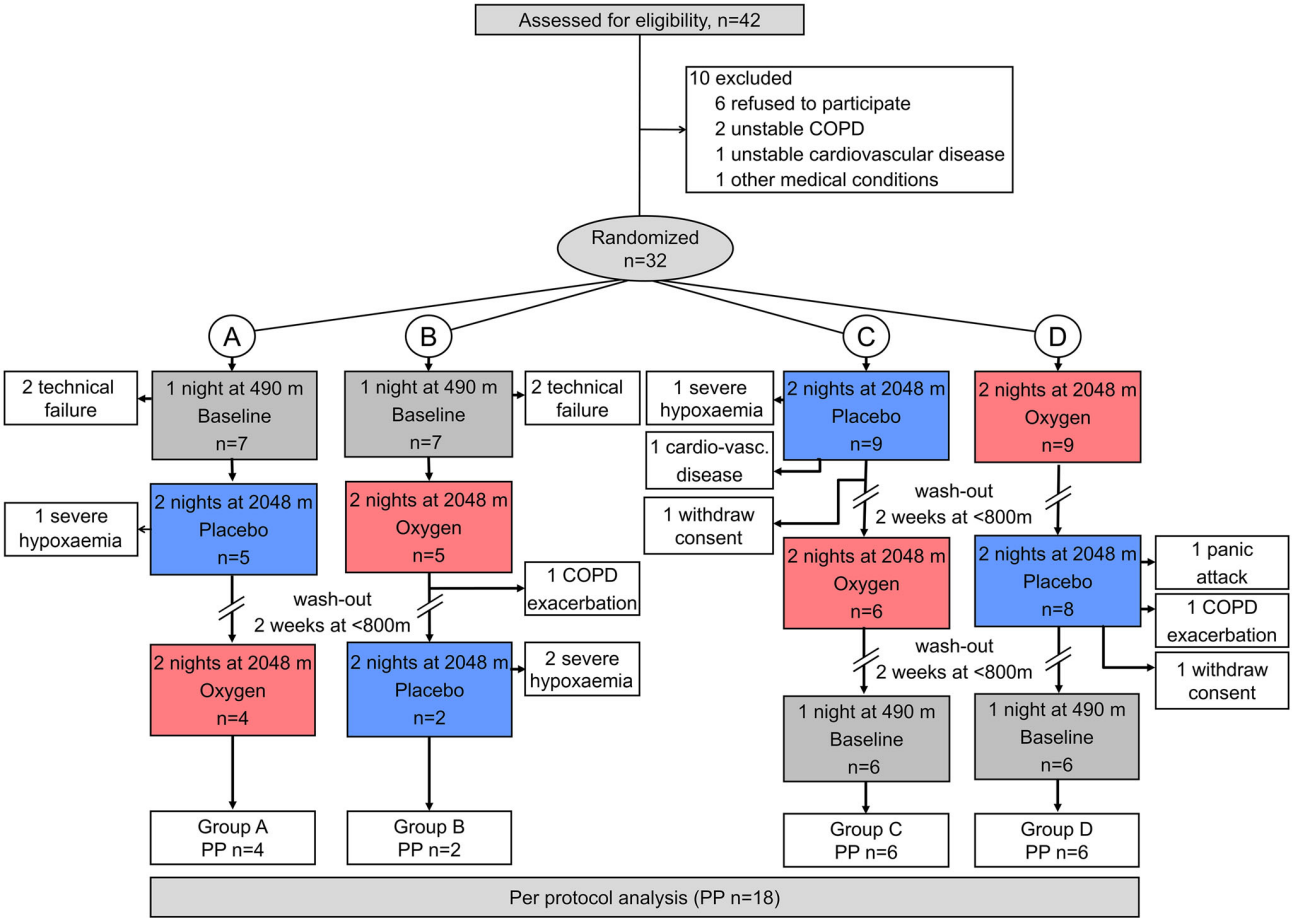
Study flow chart. COPD, chronic obstructive pulmonary disease; PP, per‐protocol.

A total of 32 patients with moderate‐to‐severe COPD were randomized for this study. At 2048 m, 31% of patients experienced an altitude‐related adverse health effect; four patients experienced severe nocturnal hypoxaemia defined as $S_{pO_{2}}$ of <75% for <30 min, two patients developed an exacerbation of their COPD, two patients wished to be relocated to low altitude owing to feeling ill, one patient experienced a panic attack, and one patient had non‐sustained ventricular tachycardia (Tan et al., 2020). Furthermore, four patients could not be included in the final analysis owing to technical failures. Therefore, 18 patients finished the crossover trial and were included in the final analysis (Figure 1). The study participants comprised 8 (44%) women and 10 (56%) men aged 65 ± 5 years with a mean FEV_1_ of 54.7% ± 13.9% predicted. In Table 1, the characteristics of patients are summarized.

**TABLE 1:** Patient characteristics of the per‐protocol population of lowland living participants with chronic obstructive pulmonary disease.

**Parameter**	**Value**
Subjects (women), *n*	18 (8)
Age, years	65 ± 5
Height, cm	169 ± 0.1
Weight, kg	73.5 ± 12
BMI, kg m^−2^	25.8 ± 4.0
Smoking status	
Current, *n* (%)	4 (22.2)
Former, *n* (%)	14 (77.8)
Smoking, pack‐years	45.2 ± 31.2
FEV_1_, % predicted	54.7 ± 13.9
FVC, % predicted	80.3 ± 17
FEV_1_/FVC, %	54.1 ± 7.3
CAT score	7.3 ± 5.1
DLCO, % predicted	72.9 ± 20.6
Comorbidities, *n* (%)	
Arterial hypertension	8 (44.4)
Coronary or cardiovascular disease	5 (27.8)
Diabetes mellitus	3 (16.7)
Dyslipidaemia	3 (16.7)
Medication, *n* (%)	
Short‐acting β‐agonists	2 (11.1)
Long‐acting β‐agonists	13 (72.2)
Short‐acting anticholinergics	1 (5.6)
Long‐acting anticholinergics	12 (66.7)
Inhaled corticosteroids	10 (55.6)
ACE inhibitors or AT antagonists	8 (44.4)
β‐Blockers	3 (16.7)
Calcium channel blockers	4 (22.2)
Diuretics	6 (33.3)
Lipid‐lowering therapy	7 (38.9)
Antidiabetic therapy	3 (16.7)
Antiplatelet aggregation therapy	7 (38.9)
Antidepressants	3 (16.7)

*Note*: Values are presented as the mean ± SD or number and percentage. Abbreviations: ACE, angiotensin‐converting enzyme; AT, angiotensin; BMI, body mass index; CAT, COPD assessment test; COPD, chronic obstructive pulmonary disease; DCLO, diffusing capacity of the lung for carbon monoxide; FEV_1_, forced expiratory volume in 1 s; FVC, forced vital capacity.

### 3.2 Resting conditions, quiet room‐air breathing

The cardio‐ and cerebrovascular outcomes at rest are displayed in Table 2. At 490 m, patients were mildly hypoxaemic but otherwise normocapnic and normotensive. Median (interquartile range) sMCAv was 45.9 (37.8; 55.4) cm^−1^ with cerebrovascular conductance and resistance index of 0.5 (0.4; 0.6) cm s^−1^ mmHg^−1^ and 2.1 (1.5; 2.5) mmHg cm^−1^ s^−1^, respectively, and a mean ± SD CTO of 60.5% ± 7.5%.

**TABLE 2:** Cardio‐ and cerebrovascular parameters at rest, breathing room air.

Parameter	490 m	2048 m, after placebo	2048 m, after NOT	Treatment effect, NOT versus placebo (95% CI)	*p*‐Value	2048 m, combined NOT and placebo
$S_{pO_{2}}$, %	93.7 ± 1.6	90.3 ± 1.6^*^	90.1 ± 1.6^*^	−0.2 (−1.1 to 0.6)	0.613	90.2 ± 1.3^*^
$P_{\mathrm{ET},CO_{2}}$, mmHg	34.1 ± 5.5	34.4 ± 5.5	35.5 ± 5.5	1.1 (−1.4 to 3.6)	0.395	35.0 ± 4.7
PtcCO_2_, mmHg	36.4 ± 5.2	38.1 ± 5.2	38.9 ± 5.2^*^	0.8 (−1.4 to 2.9)	0.492	38.5 ± 4.7^*^
Systolic BP, mmHg	131 ± 18	135 ± 18	132 ± 18	−3 (−12 to 6)	0.502	134 ± 17
Diastolic BP, mmHg	76 ± 11	78 ± 11	74 ± 11	−4 (−10 to 2)	0.155	76 ± 8
MAP, mmHg	94 ± 12	97 ± 12	94 ± 12	−4 (−10 to 2)	0.225	95 ± 8
Heart rate, beats min^−1^	72 (67; 78)	77 (73; 86)^*^	77 (73; 86)^*^	−1 (−5 to 3)	0.566	79 (73; 86)^*^
sMCAv, cm s^−1^	45.9 (37.8; 55.4)	43.1 (37.5; 48.6)	43.3 (36.6; 46.1)	0.8 (−5.9 to 7.6)	0.810	43.3 (37.1; 48.3)
CVCi, cm s^−1^ mmHg^−1^	0.5 (0.4; 0.6)	0.5 (0.4; 0.5)	0.5 (0.4; 0.5)	0 (0 to 0.1)	0.417	0.5 (0.4; 0.5)
CVRi, mmHg cm^−1^ s^−1^	2.1 (1.5; 2.5)	2.2 (2.0; 2.8)	2.2 (1.9; 2.7)	−0.2 (−0.7 to 0.4)	0.497	2.2 (1.9; 2.8)
CTO, %	60.5 ± 7.5	58.7 ± 7.5	59.0 ± 7.5	0.4 (−2.6 to 3.4)	0.806	58.9 ± 6.8

*Note*: Measured parameters are presented as the mean ± SD or median (interquartiles), with the treatment effect as mean difference (95% CI). Abbreviations: BP, blood pressure; CTO, cerebral tissue oxygenation measured by near‐infrared spectroscopy; CVCi, cerebrovascular conductance index; CVRi, cerebrovascular resistance index; HR, heart rate; MAP, mean arterial pressure measured by finger clamp technique; NOT, nocturnal oxygen therapy; $P_{\mathrm{ET},CO_{2}}$, end‐tidal partial pressure of carbon dioxide assessed by capnography; PtcCO_2_, trans‐cutaneous partial pressure of CO_2_; sMCAv, middle cerebral artery peak systolic blood flow velocity measured by transcranial Doppler ultrasound; $S_{pO_{2}}$, arterial oxygenation.

* *p *< 0.05 versus 490 m (altitude effect).

^#^ *p *< 0.05 NOT versus placebo (treatment effect). No effect detected.

At 2048 m, patients became more hypoxaemic and had a higher heart rate in comparison to 490 m (*p *< 0.05 for both comparisons). At 2048 m, median (interquartile range) sMCAv was 43.1 (37.5; 48.6) cm^−1^ in patients who had received placebo and 43.3 (36.6; 46.1) cm^−1^ in patients who had received NOT (*p* = 0.810). The mean CVCi, CVRi and MAP were not significantly different between the NOT and placebo interventions (Table 2).

### 3.3 Breathing manoeuvres

**FIGURE 2:**
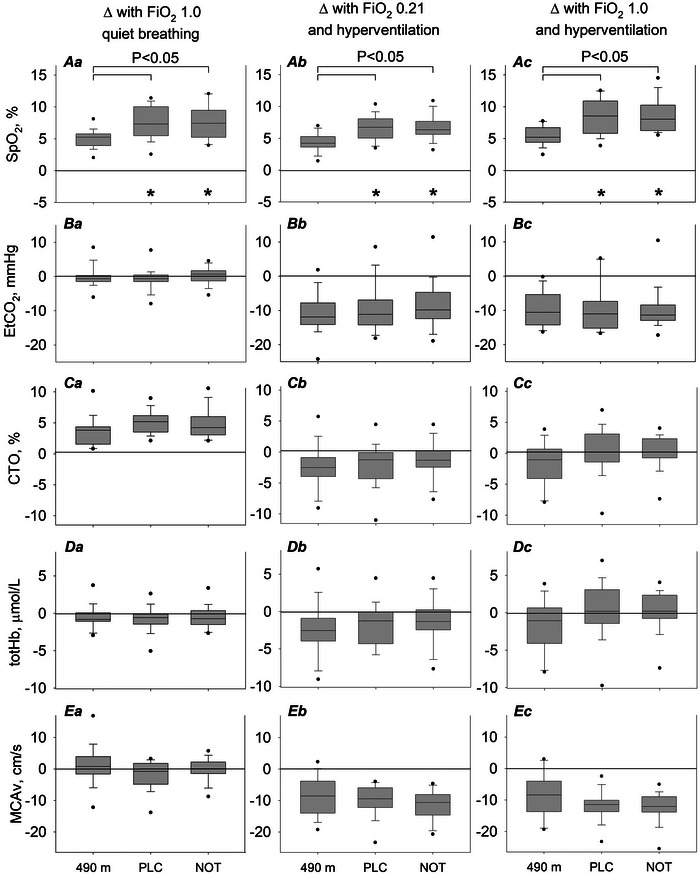
Effects of breathing manoeuvres on physiological outcomes. Changes from the pre‐manoeuvre setting are represented as bar graphs and displayed as the median (horizontal line), quartiles (boxes), 10th and 90th percentiles (whiskers) and minimum and maximum values (dots). #*p *< 0.05 versus PLC (treatment effect); *p < 0.05 versus 490 m (altitude effect). The raw dataset supporting the figure can be found in the Supporting Information. Abbreviations: CTO, cerebral tissue oxygenation; EtCO_2_, end‐tidal pressure of carbon dioxide assessed by capnography; $F_{IO_{2}}$, fraction of inhaled oxygen during the breathing manoeuvres; NOT, nocturnal oxygen therapy; PLC, placebo at night; sMCAv, middle cerebral artery peak systolic blood flow velocity measured by transcranial Doppler ultrasound; $S_{pO_{2}}$, arterial oxygen saturation measured by oximetry; totHb, total haemoglobin concentration, measured by near‐infrared spectroscopy.

In Figure 2 and Table 3, changes of measured and calculated parameters during the conducted breathing manoeuvres [quiet oxygen breathing (hyperoxia); hyperventilating ambient air (hypocapnia); and hyperventilating oxygen (hyperoxia and hypocapnia)] are summarized. The breathing manoeuvres at both altitudes (490 m; 2048 m, placebo; and 2048 m, NOT) showed consistent changes in CBF and oxygenation in comparison to pretest normal breathing at the corresponding location. These changes at 2048 m were independent of the applied nocturnal intervention (NOT or placebo; Tables S1–S3). Based on these observations, the data from the 2048 m, placebo and 2048 m, NOT interventions were combined and analysed together to assess the effects of breathing manoeuvres on CBF in COPD at 2048 versus 490 m.

**TABLE 3:** Cardio‐ and cerebrovascular responsiveness to changing blood gases.

Parameter	Oxygen at rest	Ambient air HV	Oxygen HV
Breathing manoeuvres at 490 m			
$S_{pO_{2}}$, %	+5.0 (4.1 to 5.9)^†^	+4.3 (3.5 to 5.2)^†^	+5.4 (4.5 to 6.3)^†^
$P_{\mathrm{ET},CO_{2}}$, mmHg	0 (−2.6 to 2.5)	−10.8 (−13.4 to −8.3)^†^	−9.8 (−12.4 to −7.3)^†^
Systolic BP, mmHg	−2 (−11 to 7)	−5 (−14 to −4)	−9 (−18 to 0)^†^
Diastolic BP, mmHg	0 (−6 to 6)	−1 (−7 to 4)	−0.8 (−7 to 5)
MAP, mmHg	−1 (−7 to 5)	−3 (−9 to 4)	−4 (−10 to 3)
Heart rate, beats min^−1^	−4 (−7 to −1)^†^	+5 (2 to 7)^†^	+4 (1 to 7)^†^
sMCAv, cm s^−1^	+1.0 (−5.8 to 7.8)	−8.6 (−15.4 to −1.9)^†^	−8.9 (−15.6 to −2.1)^†^
CVCi, cm s^−1^ mmHg^−1^	0 (−0.1 to 0.1)	−0.1 (−0.2 to 0)^†^	−0.1 (−0.2 to 0)^†^
CVRi, mmHg cm^−1^ s^−1^	−0.1 (−0.7 to 0.5)	+0.6 (0 to 1.1)^†^	+0.6 (0 to 1.2)^†^
CTO, %	+3.6 (0.5 to 6.7)^†^	−2.4 (−5.5 to 0.7)	−1.7 (−4.8 to 1.4)
totHb, Δµmol L^−1^	−0.6 (−1.4 to 0.3)	+0.2 (−0.7 to 1.0)	−0.7 (−1.6 to 0.1)
O_2_Hb, Δµmol L^−1^	+1.4 (0.6 to 2.3)^†^	+0.3 (−0.6 to 1.1)	+0.5 (−0.3 to 1.4)
HHb, Δµmol L^−1^	−1.9 (−2.4 to −1.5)^†^	−0.1 (−0.5 to 0.3)	−1.2 (−1.7 to −0.8)^†^
ΔsMCAv/Δ$P_{\mathrm{ET},CO_{2}}$, cm s‐1 mmHg‐1	0.5 ± 3.0	0.7 ± 3.0	0.7 ± 3.0
ΔsMCAv/Δ$S_{pO_{2}}$, cm s^−1^ %^−1^	0.2 ± 0.8	−2.0 ± 0.8	−1.7 ± 0.8
Breathing manoeuvres at 2048 m, combined groups NOT and placebo			
$S_{pO_{2}}$, %	+7.5 (6.9 to 8.1)^*^, ^†^	+6.6 (6.0 to 7.3)^*^, ^†^	+8.6 (8.0 to 9.2)^*^, ^†^
$P_{\mathrm{ET},CO_{2}}$, mmHg	−0.3 (−2.1 to 1.5)	−9.1 (−10.9 to −7.3)^†^	−9.7 (−11.5 to −7.9)^†^
Systolic BP, mmHg	0 (−6 to 7)	−5 (−11 to 2)	−6 (−12 to 1)
Diastolic BP, mmHg	+1 (−3 to 5)	−1 (−6 to 3)	0 (−5 to 4)
MAP, mmHg	0 (−4 to 5)	−3 (−7 to 2)	−2 (−7 to 2)
Heart rate, beats min^−1^	−4 (−7 to −1)^†^	+5 (2 to 7)^†^	+4 (2 to 7)^†^
sMCAv, cm s^−1^	−0.9 (−5.7 to 3.9)	−10.7 (−15.5 to −5.9)^†^	−11.9 (−16.7 to −7.1)^†^
CVCi, cm s^−1^ mmHg^−1^	0 (−0.1 to 0)	−0.1 (−0.2 to 0)^†^	−0.1 (−0.2 to −0.1)^†^
CVRi, mmHg cm^−1^ s^−1^	+0.1 (−0.3 to 0.5)	+0.7 (0.3 to 1.1)^†^	+0.9 (0.5 to 1.3)^†^
CTO, %	+5.0 (2.8 to 7.1)^†^	−1.6 (−3.7 to 0.5)	+0.3 (−1.8 to 2.4)
totHb, Δµmol L^−1^	−0.7 (−1.2 to −0.1)^†^	−0.4 (−1.0 to 0.1)	−0.6 (−1.2 to 0)^†^
O_2_Hb, Δµmol L^−1^	+2.1 (1.5 to 2.7)^†^	+0.6 (0 to 1.2)^†^	+1.6 (1.0 to 2.2)^*^, ^†^
HHb, Δµmol L^−1^	−2.8 (−3.1 to −2.5)^*^, ^†^	−1.0 (−1.3 to −0.8)^*^, ^†^	−2.2 (−2.5 to −1.9)^*^, ^†^
ΔsMCAv/Δ$P_{\mathrm{ET},CO_{2}}$, cm s^−1^ mmHg^−1^	0.3 ± 2.1	1.1 ± 2.1	0.9 ± 2.1
ΔsMCAv/Δ$S_{pO_{2}}$, cm s^−1^ %^−1^	−0.1 ± 0.8	−1.7 ± 0.8	−1.5 ± 0.8

*Note*: Given that no difference between NOT and placebo application was found on the outcomes during the displayed breathing manoeuvres at 2048 m, the two intervention groups were combined. Detailed representation of the individual breathing manoeuvres is provided in Tables S1–S3. Changes from baseline (breathing room air at rest at the corresponding location) are presented as the mean (95% CI) for measured parameters. Calculated parameters are presented as absolute values as mean ± SD. Abbreviations: CTO, cerebral tissue oxygenation; CVCi, cerebrovascular conductance index; CVRi, cerebrovascular resistance index; HHb, deoxygenated haemoglobin, measured by near‐infrared spectroscopy; HV, forced hyperventilation; MAP, mean arterial pressure measured by finger clamp technique; NOT, nocturnal oxygen therapy; O_2_Hb, oxygenated haemoglobin; $P_{\mathrm{ET},CO_{2}}$, end‐tidal partial pressure of carbon dioxide assessed by capnography; sMCAv, middle cerebral artery peak systolic blood flow velocity measured by transcranial Doppler ultrasound; $S_{pO_{2}}$, arterial oxygen saturation; totHb, total haemoglobin.

* *p *< 0.05 versus 490 m (altitude effect).

^†^ *p *< 0.05 versus pretest setting at corresponding location (breathing manoeuvre effect).

#### 3.3.1 Quiet oxygen breathing ($F_{IO_{2}}$ 1.0)

At 490 m, breathing oxygen significantly increased $S_{pO_{2}}$, CTO and O_2_Hb and decreased heart rate and HHb (Table 3). No changes were found in BP, totHb, CVCi, CVRi or sMCAv in comparison to quiet room air breathing. At 2048 m, breathing oxygen increased $S_{pO_{2}}$ and decreased HHb significantly more in comparison to 490 m. Other cardiac and cerebrovascular parameters changed in a similar manner.

#### 3.3.2 Hyperventilation with ambient air ($F_{IO_{2}}$ 0.21)

At 490 m, hyperventilation with ambient air significantly increased $S_{pO_{2}}$ and heart rate and decreased $P_{\mathrm{ET},CO_{2}}$ (Table 3). Also, CVCi increased and CVRi decreased, leading to a significant fall in mean sMCAv. Blood pressure, CTO and other parameters measured by near‐infrared spectroscopy were not altered in comparison to quiet room air breathing. At 2048 m, $S_{pO_{2}}$ increased and, additionally, HHb decreased significantly more in comparison to room air hyperventilation at 490 m. All other cardiac and cerebrovascular parameters changed in a similar manner at altitude.

#### 3.3.3 Hyperventilation while breathing oxygen ($F_{IO_{2}}$ 1.0)

At 490 m, hyperventilation while breathing oxygen significantly increased $S_{pO_{2}}$ and heart rate and decreased $P_{\mathrm{ET},CO_{2}}$, systolic BP and HHb (Table 3). The CVCi decreased significantly, and CVRi increased, leading to a reduction in sMCAv. No changes, however, were found in CTO, totHb and O_2_Hb in comparison to quiet room air breathing. At 2048 m, $S_{pO_{2}}$ increased and HHb decreased significantly more in comparison to oxygen hyperventilation at 490 m. All other cardiac and cerebrovascular parameters changed in a similar manner at altitude, except that systolic BP remained the same.

### 3.4 Head‐up tilt manoeuvre

**FIGURE 3:**
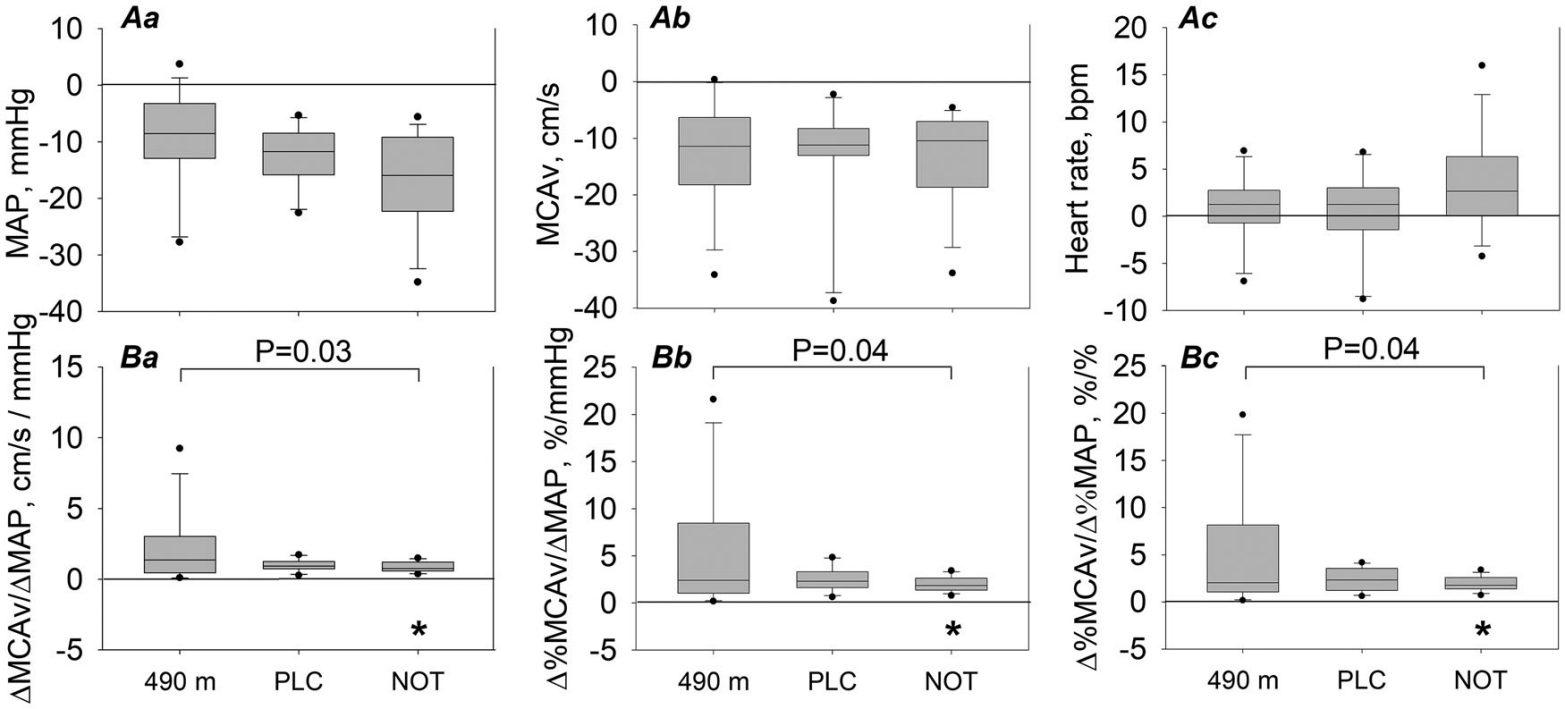
Supine‐to‐60° head‐up tilt. Changes from the pre‐manoeuvre setting (*Aa–Ac*) and absolute values following the tilt manoeuvre (*Ba–Bc*) are represented as bar graphs and displayed as the median (horizontal line), quartiles (boxes), 10th and 90th percentiles (whiskers) and minimum and maximum values (dots). #*p *< 0.05 versus PLC (treatment effect); *p < 0.05 versus 490 m (altitude effect). The raw dataset supporting the figure can be found in the Supporting Information. Abbreviations: HR, heart rate; MAP, mean arterial pressure; MCAv, middle cerebral artery peak blood flow velocity measured by transcranial Doppler ultrasound; NOT, nocturnal oxygen therapy; PLC, placebo at night.

As in the analysis of the breathing manoeuvres, the head‐up tilt manoeuvre showed consistent changes in blood pressure and reductions in sMCAv between the placebo and NOT intervention at 2048 m (Figure 3; Table S4). At 490 m, the supine‐to‐60° tilt significantly lowered systolic and mean BP (Table 4). The CVCi decreased and CVRi increased, leading to a significant reduction in sMCAv. At 2048 m, systolic, diastolic and mean arterial BP decreased significantly. The CVCi, CVRi and sMCAv were similarly influenced by the manoeuvre at altitude compared with 490 m. Indices of cerebrovascular responsiveness to changes of blood pressure (ΔsMCAv/ΔMAP, %ΔsMCAv/ΔMAP and %ΔsMCAv/%ΔMAP) were significantly reduced at 2048 compared with 490 m.

**TABLE 4:** Cardio‐ and cerebrovascular responsiveness to systemic hypotension: Supine‐to‐60° tilt, combined groups NOT and placebo in 2048 m.

Parameter	490 m	2048 m, combined NOT and placebo	*p*‐Value
Systolic BP, mmHg	−14 (−24 to −4)^†^	−19 (−26 to −11)^†^	0.461
Diastolic BP, mmHg	−6 (−12 to 0)	−10 (−15 to −6)^†^	0.238
MAP, mmHg	−10 (−17 to −4)^†^	−14 (−19 to −9)^†^	0.352
Heart rate, beats min^−1^	0 (−4 to 4)	+3 (0 to 6)^†^	0.210
sMCAv, cm s^−1^	−13.3 (−20.6 to −6.0)^†^	−15.8 (−21.2 to −10.4)^†^	0.590
CVCi, cm s^−1^ mmHg^−1^	−0.1 (−0.2 to 0)^†^	−0.1 (−0.2 to −0.1)^†^	0.792
CVRi, mmHg cm^−1^ s^−1^	+0.9 (0.3 to 1.5)^†^	+1.0 (0.6 to 1.5)^†^	0.786
ΔsMCAv/ΔMAP, cm s^−1^ mmHg^−1^	2.3 ± 1.7	0.9 ± 1.3^*^	0.007
Δ%sMCAv/ΔMAP, % mmHg^−1^	5.6 ± 4.7	2.3 ± 3.4^*^	0.013
Δ%sMCAv/Δ%MAP, % %^−1^	5.2 ± 4.2	2.2 ± 3.4^*^	0.014

*Note*: Given that no difference between NOT and placebo application was found on the outcomes during the displayed manoeuvre at 2048 m, the two intervention groups were combined. Detailed representation of the head‐up tilt manoeuvre is provided in Table S4. Changes from baseline (breathing room air at rest at the corresponding location) are presented as the mean (95% CI) for measured parameters. Calculated parameters are presented in absolute values as the mean ± SD. Abbreviations: BP, blood pressure; CVCi, cerebrovascular conductance index; CVRi, cerebrovascular resistance index; MAP, mean arterial pressure measured by finger clamp technique; NOT, nocturnal oxygen therapy; sMCAv, middle cerebral artery peak systolic blood flow velocity measured by transcranial Doppler ultrasound.

* *p *< 0.05 versus 490 m (altitude effect).

^†^ *p *< 0.05 versus pretest setting at corresponding location (tilt manoeuvre effect).

## 4 DISCUSSION

In this randomized crossover trial, we showed that an ascent to 2048 m was tolerated by the majority of patients with moderate‐to‐severe COPD (Tan et al., 2020). At 2048 m, patients suffered from daytime hypoxaemia and elevated heart rate; however, cerebrovascular determinants (i.e., CTO or sMCAv) remained unchanged in comparison to 490 m and were not altered the next day, whether patients received NOT or placebo during the night at altitude. Moreover, cerebrovascular responsiveness (ΔsMCAv/ΔMAP) to orthostatic hypotension was not deteriorated. These findings suggest that cerebrovascular homeostasis at rest is maintained at moderate altitude in COPD. Correspondingly, NOT improved nocturnal oxygenation and breathing patterns compared with placebo (Tan et al., 2020), but it did not modify determinants of cerebrovascular homeostasis at rest.

It is established that upon arrival at high altitude (>2500 m), CBF is acutely increased in healthy humans and remains elevated during the first few days at altitude (Brugniaux et al., 2007). With the progression of acclimatization, CBF gradually decreases to pre‐exposure values (Brugniaux et al., 2007). The cerebrovascular response to poikilocapnic hypoxia at altitude is guided by the cerebral vasodilatation caused by hypoxaemia, in addition to the counterbalancing vasoconstriction as an effect of hypocapnia. With the increase of CBF attributable to hypoxaemia, oxygen delivery and metabolic rate in the brain can be maintained at a relatively constant level. This was confirmed indirectly in the present study at moderate altitude, where CTO remained stable. The maintained daytime CTO is in contrast to the observed nocturnal cerebral hypoxia in these patients while sleeping (Tan et al., 2020), suggesting that cerebral protection might be dependent on sleep/wakefulness state and activity at moderate altitude in COPD. In the same patients as in the present study, Tan et al. (2020) reported that during placebo treatment at 2048 m, COPD patients had significantly lower nocturnal CTO values and elevated intermittent cerebral hypoxic events (related to sleep‐disordered breathing) in comparison to 490 m. These findings indicate that insufficient cerebral autoregulatory compensation at night was completely eliminated with NOT at 2048 m. Lower nocturnal cerebral protection has been suggested to be related to non‐rapid eye movement sleep, because cerebral autoregulation has been proposed to be inactive during non‐rapid eye movement sleep in comparison to the awake condition in COPD, and was previously observed at 2590 m (Alexandre et al., 2016; Furian et al., 2021; Meadows et al., 2004). In addition to investigating quiet resting conditions in COPD at 2048 m, Gutweniger et al. (2021) investigated submaximal exercise performance and the related exercise‐limiting factors at 2048 compared with 490 m. She revealed that the lower endurance time at 2048 compared with 490 m during a constant work‐rate exercise test at 60% of maximal work capacity was related, among other factors, to exercise‐induced cerebral hypoxia. Similar to the present study, NOT showed no beneficial effect on next‐day exercise performance. In summary, previous and present findings reveal that non‐rapid eye movement sleep stage and cardiopulmonary challenge with exercise, but not quiet resting conditions, are capable of inducing cerebral hypoxia while staying at moderate altitude with COPD.

In the present study, we investigated further the cerebrovascular responsiveness to alterations of $S_{pO_{2}}$, $P_{\mathrm{ET},CO_{2}}$ and blood pressure using breathing manoeuvres and a head‐up tilt test. With the voluntary hyperventilation manoeuvres in ambient air and hyperoxia (Table 3), we observed similar decrements in $P_{\mathrm{ET},CO_{2}}$ at both altitudes, but higher increments in $S_{pO_{2}}$ without any differences in changes in CTO and the cerebrovascular response to changes in blood gases. This was confirmed by unchanged values of ΔsMCAv/Δ$P_{\mathrm{ET},CO_{2}}$ or ΔsMCAv/Δ$S_{pO_{2}}$ at 2048 compared with 490 m. When challenging the cerebrovascular reactivity during head‐up tilting, we observed similar decrements in blood pressure and sMCAv at 2048 compared with 490 m. The significantly lower surrogate values for cerebrovascular reactivity (ΔsMCAv/ΔMAP) (Table 4) suggest even tighter and therefore better cerebrovascular reactivity in response to changes in BP at 2048 compared with 490 m in COPD patients. These findings are in line with the overall findings of this study, suggesting that the cerebrovascular homeostasis and reactivity in resting conditions are maintained at moderate altitude in COPD. At altitude, no further COPD studies are available to compare with the present results. At low altitude, Hoiland et al. (2018) found the cerebral vasculature in chronic hypoxaemic COPD of moderate to very severe grades (FEV_1 _= 33% predicted, $S_{pO_{2}}$ = 91%) to be insensitive to oxygen, because no constriction in response to oxygen supplementation was observed. This circumstance can be viewed from a positive perspective (Ogoh, 2018), because it leads to elevated cerebral oxygen delivery and neurovascular coupling in those patients. These reported findings were confirmed in our study (Table 3). However, when COPD patients were more hypoxaemic at 2048 m, we observed with hyperoxic gas breathing a significant decrease in totHb, a surrogate for total blood volume, and cerebrovascular vasoconstriction. However, sMCAv remained unchanged despite a decrement in totHb.

### 4.1 Limitations

For this subproject, no sample size estimation has been performed. Given that 18 patients completed all measurements in a randomized crossover design, a minimum effect size of 0.67 would have been detectable (two‐sided α level of 0.05 and power of 80%). The non‐invasive techniques used to assess cerebrovascular characteristics, transcranial Doppler and near‐infrared spectroscopy, estimate surrogates of CBF and cerebral blood volume based on the assumption that the diameter of the insonated middle cerebral artery remains unchanged. This assumption has been shown to be correct up to altitudes of 4000–5000 m (Wilson et al., 2011), suggesting that no change in middle cerebral artery diameter in our COPD patients at 2048 m can be expected. It must be noted that any influence on vascular tone and diameter via sympathetic activity influences flow and has not been quantified and corrected. Furthermore, the measured values during the orthostatic challenge have not been corrected to the arterial partial pressure of CO_2_.

## 5 CONCLUSION

Based on these findings from a randomized clinical trial, we conclude that an ascent to moderate altitude does not impair resting cerebrovascular function, which might even be improved during the first days at moderate altitude in response to blood pressure changes in patients with moderate‐to‐severe COPD. Although cerebrovascular homeostasis and reactivity at moderate altitude remain unaffected by NOT, NOT had several other beneficial effects on nocturnal indices and the incidence of altitude‐related adverse health effects (Tan et al., 2020).

## AUTHOR CONTRIBUTIONS

Tsogyal D. Latshang, Sayaka S. Aeschbacher, Stefanie Ulrich, Konrad E. Bloch and Michael Furian contributed to the conception and design of the work. All authors contributed to the acquisition, analysis or interpretation of data for the work. Dominic Gilliand and Michael Furian drafted the article and all authors critically revised the final draft for important intellectual content. Michael Furian confirms that all authors approved the final version of the manuscript; agree to be accountable for all aspects of the work in ensuring that questions related to the accuracy or integrity of any part of the work are appropriately investigated and resolved; and all persons designated as authors qualify for authorship, and all those who qualify for authorship are listed.

## CONFLICT OF INTEREST

None declared.

## Supporting information



Supporting Information

## Data Availability

The medical data used in this study are accessible to researchers with an approved research project that meets the necessary ethical and institutional requirements of Switzerland. To request the raw data, please contact michael.furian@usz.ch
