# Supplementary material for: Effect of moderate altitude and nocturnal oxygen therapy on cerebrovascular function in patients with COPD: A randomized, crossover trial at 2048 m
Source: Exp Physiol. 2025 Jul 22;111(2):527–38. doi: 10.1113/EP093003 (PMC12857471; doi:10.1113/EP093003)
Supplement: Supplementary file 1 — Supporting Information [file EPH-111-527-s001.docx]

**Supplement to:**

**Effect of moderate altitude and nocturnal oxygen therapy on cerebrovascular function in patients with COPD. A randomized, cross-over trial at 2048 m**

**Running head:** Oxygen therapy in COPD at altitude

Dominic Gilliand^1^, Tsogyal D. Latshang^1^, Sayaka S. Aeschbacher^1^, Fabienne Huber^1^, Deborah Flueck^1^, Mona Lichtblau^1^, Stefanie Ulrich^1^, Elisabeth D. Hasler^1^, Philipp M. Scheiwiller^1^, Julian Müller^1^, Silvia Ulrich^1^, Konrad E. Bloch^1^, Michael Furian^1,2^

^1^Department of Respiratory Medicine, University Hospital of Zurich, Zurich, Switzerland

^2^Research Department, Swiss University of Traditional Chinese Medicine, Bad Zurzach, Switzerland

**Funding**

The study was supported by the Swiss National Science Foundation (143875) and Lunge Zurich. Siemens Health Engineers provided some equipment for the study.

**Correspondence**

Michael Furian, Dr. sc. ETH

University Hospital Zurich

Pulmonology Department

Raemistrasse 100

8092 Zurich

Michael.furian@usz.ch

| **Table S1. Effects of applying NOT vs. placebo on next day cardio- and cerebrovascular indices in patients with COPD at altitude –response to breathing oxygen F_i_O_2_ 1.0 (hyperoxia)** | | | | | |
| --- | --- | --- | --- | --- | --- |
|  | **490 m** | **2048 m - placebo** | **2048 m - NOT** | **Treatment effect** | **P value** |
| **SpO_2_, %** | +5.0 (4.1 to 5.9) ^¶^ | +7.5 (6.6 to 8.3) ^¶^* | +7.6 (6.7 to 8.5) ^¶^* | +0.1 (-1.1 to 1.3) | 0.862 |
| **PetCO_2_, mmHg** | 0 (-2.6 to 2.5) | -0.7 (-3.2 to 1.8) | +0.2 (-2.3 to 2.7) | +0.9 (-2.7 to 4.4) | 0.624 |
| **Systolic BP, mmHg** | -2 (-11 to 7) | -1 (-10 to 8) | +1 (-8 to 10) | +2 (-11 to 15) | 0.745 |
| **Diastolic BP, mmHg** | 0 (-6 to 5) | +1 (-5 to 6) | 0 (-5 to 6) | 0 (-8 to 8) | 0.930 |
| **MAP, mmHg** | -1 (-7 to 5) | 0 (-6 to 6) | +1 (-5 to 7) | 0 (-8 to 9) | 0.913 |
| **Heart rate, bpm** | -4 (-7 to 0) | -4 (-8 to 0) ^¶^ | -4 (-8 to 0) ^¶^ | 0 (-5 to 5) | 0.967 |
| **sMCAv, cm s^-1^** | +1.0 (-5.8 to 7.8) | -1.7 (-8.5 to 5.0) | 0 (-6.8 to 6.8) | +1.7 (-7.9 to 11.3) | 0.725 |
| **CVCi, cm s^-1^ mmHg^-1^** | 0 (-0.1 to 0.1) | 0 (-0.1 to 0.1) | 0 (-0.1 to 0.1) | 0 (-0.1 to 0.1) | 0.714 |
| **CVRi, mmHg cm^-1^ s^-1^** | -0.1 (-0.7 to 0.5) | +0.2 (-0.3 to 0.8) | 0 (-0.5 to 0.6) | -0.2 (-1.0 to 0.6) | 0.601 |
| **CTO, %** | +3.6 (0.5 to 6.7) ^¶^ | +5.1 (2.1 to 8.0) ^¶^ | +4.9 (1.9 to 7.9) ^¶^ | -0.2 (-4.4 to 4.1) | 0.938 |
| **totHb, Δμmol L^-1^** | -0.6 (-1.4 to 0.3) | -0.7 (-1.6 to 0.1) | -0.6 (-1.4 to 0.2) | +0.2 (-1.0 to 1.3) | 0.767 |
| **O_2_Hb, Δμmol L^-1^** | +1.4 (0.6 to 2.3) ^¶^ | +2.0 (1.2 to 2.9) ^¶^ | +2.2 (1.4 to 3.0) ^¶^ | +0.2 (-1.0 to 1.3) | 0.799 |
| **HHb, Δμmol L^-1^** | -1.9 (-2.4 to -1.5) ^¶^ | -2.8 (-3.2 to -2.4) ^¶^* | -2.8 (-3.2 to -2.4) ^¶^* | 0 (-0.6 to 0.6) | 0.999 |
| **ΔsMCAv/ ΔPetCO_2_, cm s^-1^ mmHg-^1^** | 0.5 ± 0.7 | 0.4 ± 0.7 | 0.2 ± 0.7 | -0.2 (-2.1 to 1.7) | 0.837 |
| **ΔsMCAv/ ΔSpO_2_, cm s^-1^ %^-1^** | 0.2 ± 0.2 | -0.2 ± 0.2 | 0.1 ± 0.2 | +0.3 (-0.3 to 0.9) | 0.354 |
| Changes from baseline (breathing room air at rest at corresponding location) are presented as mean (95% CI) for measured parameters. Calculated parameters are presented in absolute values as mean ± SD, and the treatment effect as mean difference (95% CI). NOT, nocturnal oxygen therapy; SpO_2_, arterial oxygen saturation; PetCO_2_, end-tidal partial pressure of carbon dioxide assessed by capnography; MAP, mean arterial pressure measured by finger clamp technique; sMCAv, middle cerebral artery peak systolic blood flow velocity measured by transcranial Doppler ultrasound; CVCi, cerebrovascular conductance index; CVRi, cerebrovascular resistance index; CTO, cerebral tissue oxygenation measured by near-infrared spectroscopy; totHb, total hemoglobin; O_2_Hb, oxygenated hemoglobin; HHb, deoxygenated hemoglobin.  ^¶^ P<0.05 vs. rest at corresponding location (breathing maneuver effect)  * P<0.05 vs. change at 490 m (altitude effect)  # P<0.05 NOT vs. PLC (treatment effect). No effect detected. | | | | | |

| **Table S2. Effects of applying NOT vs. placebo on next day cardio- and cerebrovascular indices in patients with COPD at altitude – response to ambient air hyperventilation F_i_O_2_ 0.21 (hypocapnia)** | | | | | |
| --- | --- | --- | --- | --- | --- |
|  | **490 m** | **2048 m - placebo** | **2048 m - NOT** | **Treatment effect** | **P value** |
| **SpO_2_, %** | +4.4 (3.5 to 5.2) ^¶^ | +6.6 (5.8 to 7.5) ^¶^* | +6.6 (5.8 to 7.5) ^¶^* | 0 (-1.2 to 1.2) | 0.996 |
| **PetCO_2_, mmHg** | -10.8 (-13.3 to -8.3) ^¶^ | -9.6 (-12.1 to -7.0) ^¶^ | -8.7 (-11.2 to -6.2) ^¶^ | +0.8 (-2.7 to 4.4) | 0.644 |
| **Systolic BP, mmHg** | -5 (-14 to 4) | -8 (-17 to 1) | -2 (-11 to 8) | +6 (-7 to 19) | 0.342 |
| **Diastolic BP, mmHg** | -1 (-7 to 4) | -3 (-8 to 3) | 0 (-6 to 6) | +3 (-5 to 11) | 0.488 |
| **MAP, mmHg** | -3 (-9 to 3) | -5 (-11 to 2) | -1 (-7 to 5) | +4 (-5 to 13) | 0.365 |
| **Heart rate, bpm** | +6 (3 to 10) ^¶^ | +4 (1 to 8) ^¶^ | +5 (1 to 8) ^¶^ | +1 (-4 to 6) | 0.807 |
| **sMCAv, cm s^-1^** | -8.6 (-15.4 to -1.9) ^¶^ | -10.0 (-16.7 to -3.2) ^¶^ | -11.5 (-18.3 to -4.7) ^¶^ | -1.6 (-11.1 to 8.0) | 0.750 |
| **CVCi, cm s^-1^ mmHg^-1^** | -0.1 (-0.2 to 0.0) ^¶^ | -0.1 (-0.2 to 0.0) ^¶^ | -0.1 (-0.2 to 0.0) ^¶^ | 0.0 (-0.1 to 0.1) | 0.532 |
| **CVRi, mmHg cm^-1^ s^-1^** | +0.6 (0 to 1.1) ^¶^ | +0.6 (0.0 to 1.1) ^¶^ | +0.8 (0.3 to 1.4) ^¶^ | +0.2 (-0.6 to 1.0) | 0.553 |
| **CTO, %** | -2.4 (-5.5 to 0.7) | -1.8 (-4.8 to 1.2) | -1.3 (-4.3 to 1.7) | +0.5 (-3.7 to 4.7) | 0.817 |
| **totHb, Δμmol L^-1^** | +0.2 (-0.7 to 1.0) | -0.5 (-1.3 to 0.3) | -0.4 (-1.2 to 0.4) | +0.1 (-1.1 to 1.2) | 0.867 |
| **O_2_Hb, Δμmol L^-1^** | +0.3 (-0.6 to 1.2) | +0.6 (-0.3 to 1.4) | +0.6 (-0.2 to 1.5) | 0.0 (-1.1 to 1.2) | 0.951 |
| **HHb, Δμmol L^-1^** | -0.1 (-0.6 to 0.3) | -1.1 (-1.5 to -0.7) ^¶^* | -1.0 (-1.4 to -0.6) ^¶^* | 0.0 (-0.5 to 0.6) | 0.879 |
| **ΔsMCAv/ ΔPetCO_2_, cm s^-1^ mmHg^-1^** | 0.7 ± 0.7 | 0.6 ± 0.7 | 1.6 ± 0.7 | +1.0 (-0.9 to 3.0) | 0.306 |
| **ΔsMCAv/ ΔSpO_2_, cm s^-1^ %^-1^** | -2.0 ± 0.3 | -1.6 ± 0.3 | -1.8 ± 0.3 | -0.2 (-0.8 to 0.4) | 0.593 |

| Changes from baseline (breathing room air at rest at corresponding location) are presented as mean (95% CI) for measured parameters. Calculated parameters are presented in absolute values as mean ± SD, and the treatment effect as mean difference (95% CI). NOT, nocturnal oxygen therapy; SpO_2_, arterial oxygen saturation; PetCO_2_, end-tidal partial pressure of carbon dioxide assessed by capnography; MAP, mean arterial pressure measured by finger clamp technique; sMCAv, middle cerebral artery peak systolic blood flow velocity measured by transcranial Doppler ultrasound; CVCi, cerebrovascular conductance index; CVRi, cerebrovascular resistance index; CTO, cerebral tissue oxygenation measured by near-infrared spectroscopy; totHb, total hemoglobin; O_2_Hb, oxygenated hemoglobin; HHb, deoxygenated hemoglobin.  ^¶^ P<0.05 vs. rest at corresponding location (breathing maneuver effect)  * P<0.05 vs. change at 490 m (altitude effect)  # P<0.05 NOT vs. PLC (treatment effect). No effect detected. |
| --- |

| **Table S3. Effects of applying NOT vs. placebo on next day cardio- and cerebrovascular indices in patients with COPD at altitude – response to oxygen hyperventilation F_i_O_2_ 1.0 (hyperoxia and hypocapnia)** | | | | | |
| --- | --- | --- | --- | --- | --- |
|  | **490 m** | **2048 m - placebo** | **2048 m - NOT** | **Treatment effect** | **P value** |
| **SpO_2_, %** | +5.4 (4.5 to 6.2) ^¶^ | +8.5 (7.7 to 9.4) ^¶^* | +8.7 (7.8 to 9.5) ^¶^* | +0.2 (-1.1 to 1.4) | 0.797 |
| **PetCO_2_, mmHg** | -9.8 (-12.3 to -7.3) ^¶^ | -9.7 (-12.2 to 7.2) ^¶^ | -9.8 (-12.3 to -7.3) ^¶^ | -0.1 (-3.7 to 3.5) | 0.956 |
| **Systolic BP, mmHg** | -9 (-18 to 0) ^¶^ | -7 (-17 to 2) | -4 (-13 to 5) | +4 (-9 to 16) | 0.594 |
| **Diastolic BP, mmHg** | -1 (-6 to 5) | 0 (-6 to 5) | -1 (-6 to 5) | -1 (-8 to 7) | 0.900 |
| **MAP, mmHg** | -4 (-10 to 2) | -3 (-9 to 3) | -2 (-8 to 4) | +1 (-8 to 9) | 0.849 |
| **Heart rate, bpm** | +5 (1 to 9) ^¶^ | +4 (1 to 8) ^¶^ | +4 (0 to 8) ^¶^ | 0 (-5 to 5) | 0.956 |
| **MCAv, cm s^-1^** | -8.9 (-15.6 to -2.1) ^¶^ | -11.7 (-18.5 to -4.9) ^¶^ | -12.2 (-19.0 to -5.4) ^¶^ | -0.5 (-10.1 to 9.1) | 0.915 |
| **CVCi, cm s^-1^ mmHg^-1^** | -0.1 (-0.2 to 0.0) ^¶^ | -0.1 (-0.2 to 0.0) ^¶^ | -0.1 (-0.2 to 0.0) ^¶^ | 0 (-0.1 to 0.1) | 0.849 |
| **CVRi, mmHg cm^-1^ s^-1^** | +0.6 (0.0 to 1.1) ^¶^ | +1.0 (0.4 to 1.5) ^¶^ | +0.8 (0.2 to 1.4) ^¶^ | -0.2 (-1.0 to 0.6) | 0.655 |
| **CTO, %** | -1.7 (-4.8 to 1.4) | +0.4 (-2.6 to 3.4) | +0.2 (-2.8 to 3.2) | -0.2 (-4.4 to 4.0) | 0.930 |
| **totHb, Δμmol L^-1^** | -0.7 (-1.6 to 0.1) | -0.6 (-1.4 to 0.2) | -0.6 (-1.4 to 0.2) | 0 (-1.1 to 1.1) | 0.984 |
| **O_2_Hb, Δμmol L^-1^** | +0.5 (-0.3 to 1.3) | +1.6 (0.8 to 2.4) ^¶^ | +1.6 (0.7 to 2.4) ^¶^ | 0 (-1.2 to 1.1) | 0.943 |
| **HHb, Δμmol L^-1^** | -1.2 (-1.7 to -0.8) ^¶^ | -2.2 (-2.6 to -1.8) ^¶^* | -2.2 (-2.6 to -1.8) ^¶^* | 0 (-0.5 to 0.6) | 0.896 |
| **ΔMCAv/ ΔPetCO_2_, cm s^-1^ mmHg^-1^** | 0.7 ± 0.7 | 0.7 ± 0.7 | 1.1 ± 0.7 | +0.3 (-1.6 to 2.3) | 0.738 |
| **ΔMCAv/ ΔSpO_2_, cm s^-1^ %^-1^** | -1.7 ± 0.3 | -1.5 ± 0.3 | -1.5 ± 0.3 | 0.0 (-0.6 to 0.6) | 0.960 |
| Changes from baseline (breathing room air at rest at corresponding location) are presented as mean (95% CI) for measured parameters. Calculated parameters are presented in absolute values as mean ± SD, and the treatment effect as mean difference (95% CI). NOT, nocturnal oxygen therapy; SpO_2_, arterial oxygen saturation; PetCO_2_, end-tidal partial pressure of carbon dioxide assessed by capnography; MAP, mean arterial pressure measured by finger clamp technique; MCAv, middle cerebral artery peak blood flow velocity measured by transcranial Doppler ultrasound; CVCi, cerebrovascular conductance index; CVRi, cerebrovascular resistance index; CTO, cerebral tissue oxygenation measured by near-infrared spectroscopy; totHb, total hemoglobin; O_2_Hb, oxygenated hemoglobin; HHb, deoxygenated hemoglobin.  ^¶^ P<0.05 vs. rest at corresponding location (breathing maneuver effect)  * P<0.05 vs. change at 490 m (altitude effect)  # P<0.05 NOT vs. PLC (treatment effect). No effect detected. | | | | | |

| **Table S4. Effects of applying NOT vs. placebo on next day cardio- and cerebrovascular indices in patients with COPD at altitude – response to a supine-to-60° head-up-tilt (orthostatic hypotension)** | | | | | |
| --- | --- | --- | --- | --- | --- |
|  | **490 m** | **2048 m - placebo** | **2048 m - NOT** | **Treatment effect** | **P value** |
| **Systolic BP, mmHg** | -14 (-24 to -4) ^¶^ | -17 (-28 to -7) ^¶^ | -19 (-29 to -9) ^¶^ | -2 (-17 to 12) | 0.790 |
| **Diastolic BP, mmHg** | -6 (-12 to 0) | -8 (-15 to -2) ^¶^ | -12 (-18 to -6) ^¶^ | -4 (-12 to 6) | 0.462 |
| **MAP, mmHg** | -10 (-17 to -4) ^¶^ | -12 (-19 to -5) ^¶^ | -15 (-22 to -9) ^¶^ | -3 (-13 to 7) | 0.540 |
| **Heart rate, bpm** | 0 (-4 to 4) | +2 (-2 to 6) | +4 (0 to 8) (0.052) | +2 (-4 to 8) | 0.523 |
| **MCAv, cm s^-1^** | -13.3 (-20.5 to -6.0) ^¶^ | -16.8 (-24.7 to -9.0) ^¶^ | -14.9 (-22.4 to -7.5) ^¶^ | -1.9 (-8.9 to 12.7) | 0.605 |
| **CVCi, cm s^-1^ mmHg^-1^** | -0.1 (-0.2 to 0) ^¶^ | -0.1 (-0.2 to 0) ^¶^ | -0.1 (-0.2 to 0) ^¶^ | 0 (-0.1 to 0.2) | 0.991 |
| **CVRi, mmHg cm^-1^ s^-1^** | +0.9 (0.3 to 1.5) ^¶^ | +1.4 (0.7 to 2.0) ^¶^ | +0.7 (0.1 to 1.3) ^¶^ | -0.6 (-1.5 to 0.3) | 0.166 |
| **ΔMCAv/ΔMAP, cm s^-1^ mmHg^-1^** | 2.3 ± 0.7 | 1.0 ± 0.1 (p=0.060) | 0.9 ± 0.1 * (p=0.034) | -0.2 (-0.5 to 0.1) | 0.310 |
| **Δ%MCAv / ΔMAP, % mmHg^-1^** | 5.6 ± 1.7 | 2.6 ± 0.4 (p=0.090) | 2.0 ± 0.2 * (p=0.040) | -0.6 (1.4 to 0.3) | 0.194 |
| **Δ%MCAv / Δ%MAP** | 5.2 ± 1.6 | 2.5 ± 0.4 (p=0.098) | 1.9 ± 0.2 * (p=0.043) | -0.6 (-1.4 to 0.2) | 0.166 |
| Changes from baseline (breathing room air at rest at corresponding location) are presented as mean (95% CI) for measured parameters. Calculated parameters are presented in absolute values as mean ± SD, and the treatment effect as mean difference (95% CI). NOT, nocturnal oxygen therapy; BP, blood pressure; MAP, mean arterial pressure measured by finger clamp technique; MCAv, middle cerebral artery peak blood flow velocity measured by transcranial Doppler ultrasound; CVCi, cerebrovascular conductance index; CVRi, cerebrovascular resistance index.  ^¶^ P<0.05 vs. during rest at corresponding location (tilt maneuver effect)  * P<0.05 vs. 490 m (altitude effect)  # P<0.05 NOT vs. PLC (treatment effect). No effect detected. | | | | | |
